# Corneal Decompensation after Selective Laser Trabeculoplasty

**DOI:** 10.1155/2014/851971

**Published:** 2014-07-01

**Authors:** Ahmet Ozkok, Nevbahar Tamcelik, Didar Ucar Comlekoglu, Guzin Iskeleli

**Affiliations:** Ophthalmology Department, Cerrahpasa School of Medicine, Istanbul University, Kocamustafapasa Cd. No. 53, Cerrahpasa, Fatih, 34098 Istanbul, Turkey

## Abstract

A 64-year-old Caucasian man referred for decreased vision after selective laser trabeculoplasty (SLT). Slit lamp examination revealed diffuse corneal edema. Despite intensive topical treatment including steroids, corneal edema did not resolve; on the contrary, it advanced to bullous keratopathy. Corneal edema after SLT is an exceptionally rare complication and in all of the previous reports edema resolved with medical treatment. To the best of our knowledge, this is the first report presenting persistent corneal edema after SLT.

## 1. Introduction

Selective laser trabeculoplasty (SLT) is a widely accepted treatment modality to decrease intraocular pressure (IOP). It uses a 532 nm frequency-doubled, Q-switched Nd:YAG laser, which delivers a relatively low energy, large spot, and very brief pulse to trabecular meshwork [1]. SLT has gained wide-spread acceptance because of its comparable efficacy to argon laser trabeculoplasty and its minimal destructive effects on tissues neighboring the trabecular meshwork [2].

Complication rate of SLT is reported to be pretty low. Known complications include anterior chamber inflammation, choroidal effusion, corneal edema, ocular discomfort, and IOP elevation [3–5]. To our knowledge, there are 2 previous reports including 4 cases of post-SLT corneal edema in the literature [4, 5]. All these case reports report conservative management of corneal edema and subsequent resolution. In the present study, we describe a case of post-SLT corneal edema which did not resolve with medical treatment.

## 2. Case Report

A 64-year-old Caucasian man with primary open-angle glaucoma (POAG) and corneal edema was referred to our clinic. According to the patient's medical chart, he had had phacoemulsification and trabeculectomy operations in his right eye 4 and 3 years ago, respectively. After the failure of trabeculectomy, the patient underwent SLT (80 shots, 1,0 mJ/spot, 330 degrees) in his right eye. At the time of the treatment, the patient was on a fixed combination of topical timolol + brimonidine and topical travaprost. Pre-SLT best corrected distance visual acuity (BCVA), IOP, and central corneal thickness were 20/50, 22 mmHg, and 519 microns, respectively. Nearly total cupping of optic nerve was also reported (C/D:0.9). Topical prednisolone 1% drop (Pred Forte; Allergan, Inc., Irvine, CA) TID was started after SLT for 3 days. Mild anterior chamber inflammation was noticed and IOP was reported to be 21 mmHg on the following day. According to the patient's history, his visual acuity started to decrease at about 1 week postoperatively; however, he did not visit his physician until 2 weeks postoperatively. At 2 weeks after SLT, visual acuity and IOP in the right eye were 20/400 and 19 mmHg, respectively. Corneal sensitivity was checked with cotton-wisp test and it was symmetric in both eyes. Moderate localized central corneal edema was also reported in the right eye at that examination.

**Figure 1:**
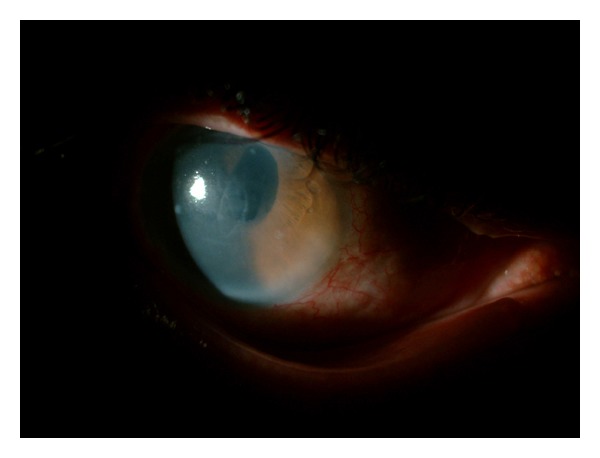
Diffuse corneal edema on slit-lamp examination of the patient's right eye.

Intensive topical treatment, including steroid and hyperosmolar sodium chloride drops, was started. At the exam 6 weeks after SLT, visual acuity and IOP were light perception and 28 mmHg, respectively. Diffuse corneal edema was also reported. At 8 weeks after SLT, visual acuity and IOP were hand motion and 9 mmHg, respectively. Diffuse corneal edema was also reported. At 12 weeks after SLT, corneal edema was still not resolved and the patient was referred to our clinic. At our examination, visual acuity was hand motion, IOP was 14 mmHg, and there was diffuse corneal edema (Figure 1). We continued intensive medical treatment but the corneal edema did not resolve; on the contrary, it progressed to bullous keratopathy. The patient did not want to have a keratoplasty operation. Since the patient had end-stage glaucoma, we did not strongly suggest him to have a keratoplasty operation. Currently, he is on therapeutic contact lens and topical treatment including a fixed combination of timolol + brimonidine along with artificial tear drop and hyperosmolar sodium chloride.

## 3. Discussion

Corneal edema after SLT is an extremely rare complication. To our knowledge, this complication has only been reported twice in the literature [4, 5]. Both reports included 2 cases, both of which resolved with medical treatment. Features of previously reported cases and the current case are summarized together in Table 1.

The etiology of post-SLT corneal edema is not exactly known. Possible culprits are postoperative anterior chamber inflammation, reactivation of latent herpes simplex virus (HSV) infection, toxicity of residual alcohol on the goniolens, and direct laser damage to the cornea [4, 5].

Guzey et al. reported that SLT increases free oxygen radicals and decreases antioxidant enzymes of the aqueous humor in rabbits [6]. Corneal endothelial cells are reported to be susceptible to oxidative stress. SLT-induced increased oxidative stress of anterior chamber may have a potential to cause corneal endothelial damage.

In a recently published study, White et al. investigated corneal effects of SLT in 10 consecutive routinely treated patients by using specular microscopy and* in vivo* confocal microscopy before and after SLT [7]. They found that SLT caused acute transient corneal endothelial changes in most patients, while having no impact on corneal endothelial cell count and visual acuity.

The number of shots in our case was relatively high, which possibly lead to an excessive energy uptake by the angle structures. This may be an etiological factor for the corneal endothelial damage.

One may speculate that, since our patient had a history of previous cataract and trabeculectomy surgeries, the patient's corneal endothelium may have already been damaged before SLT. Because corneal endothelial cell count is not measured before SLT, we cannot state that the corneal endothelium was totally healthy before the procedure. But, the expression of the patient, as well as the documented visual acuity and biomicroscopy data before SLT, indicates a corneal endothelium which is healthy enough to keep the cornea transparent. Therefore, in our case, SLT may be the last straw.

Another possible reason for corneal edema after SLT is reactivation of latent HSV infection. But the patient did not have a history of herpes labialis or herpetic eye disease. According to his chart, he had been tested for corneal sensitivity with cotton-wisp test which was reported to be symmetric in both eyes at the visit 2 weeks after SLT.

In conclusion, many studies have shown that SLT is a safe treatment modality. Although very rare, it may have a potential to damage corneal endothelial cells. In particular, in patients with known corneal endothelial problems, being aware of the potential side effect and taking preventive intraoperative and postoperative measures would be most appropriate.

## Figures and Tables

**Table 1:** Features of previously reported cases and the current case.

Report	Case	Age/sex/eye	Laser parameters	Time of corneal edema	Treatment	Resolve	VA (pre-SLT and post-SLT)
Moubayed et al. [4]	1	60/F/OS	50 shots, 360°, 0.9-1 mJ/spot	1 week	Steroid (T), NaCl (T), and valacyclovir (O)	4 months	20/25 and 20/25
	2	54/F/OD	60 shots OD, 360°, 0.8 mJ/spot	1 week	Steroid (T)	2 months	20/20 and 20/20

Regina et al. [5]	1	55/F/OS	100 shots, 360°, 1.0 J/spot,	1 day	Steroid (T)	3 weeks	20/20 and 20/20
	2	57/F/OD	104 shots, 360°, 0.9 mJ/spot	2 days	Steroid (T)	3 months	20/20 and 20/20

Current case	1	64/M/OD	80 shots, 330°1.0 mJ/spot	1 week	Steroid (T) and NaCl (T)	Not resolved (17 months)	20/50 and HM
